# When to write a neurology case report

**DOI:** 10.1186/s13256-016-0868-9

**Published:** 2016-04-06

**Authors:** Richard A. Rison, Jennifer Kelly Shepphird, Said R. Beydoun

**Affiliations:** 1 University of Southern California Keck School of Medicine, Los Angeles County Medical Center, Medical Director PIH Health Hospital-Whittier Stroke Program, Medical Director PIH Health Hospital-Whittier Non-Invasive Vascular Laboratory, Neurology Consultants Medical Group, 12401 Washington Boulevard, Whittier, CA 90602 USA; 2 JKS Science & Medical Writing, Los Angeles, CA USA; 3 Keck School of Medicine, University of Southern California, Los Angeles County Medical Center, 1520 San Pablo Street, Suite 3000, Los Angeles, CA 90033 USA

## Abstract

Case report publication has seen a resurgence in recent years as awareness of the value of case reports in clinical medicine has grown. Not all areas of medical research are amenable to large clinical trials. Many topics are better addressed by more detailed descriptions of multi-factorial components that contribute to outcomes, and these are areas where case reports shine. Determining the suitability of a case for publication requires background research and discussion. Writing a case or series reinforces many aspects of the medical training process, and house staff are encouraged to research, write, and submit reports. The medical community benefits in many ways from case reports, from improving individual patient care to guiding future research directions.

Daily interactions with patients and frequent exchanges with colleagues over time build a physician’s clinical knowledge base. This competence must be passed on through generations in efforts to achieve better patient care. Patient encounters represent the front lines of medical research. A physician’s responsibility to record observations and share new knowledge gained from these experiences forms a tradition in medicine that dates back to antiquity. It remains essential to the art of medicine and care of patients today [1].

Sir William Osler, a Canadian physician and founding professor of Johns Hopkins Hospital, created the first residency program for physicians and devoted himself to the training of new physicians (Fig. 1). As a prolific author and ardent student of medical history, Osler stated, “Always note and record the unusual…. Publish it. Place it on permanent record as a short, concise note. Such communications are always of value” [2].

**Fig. 1:**
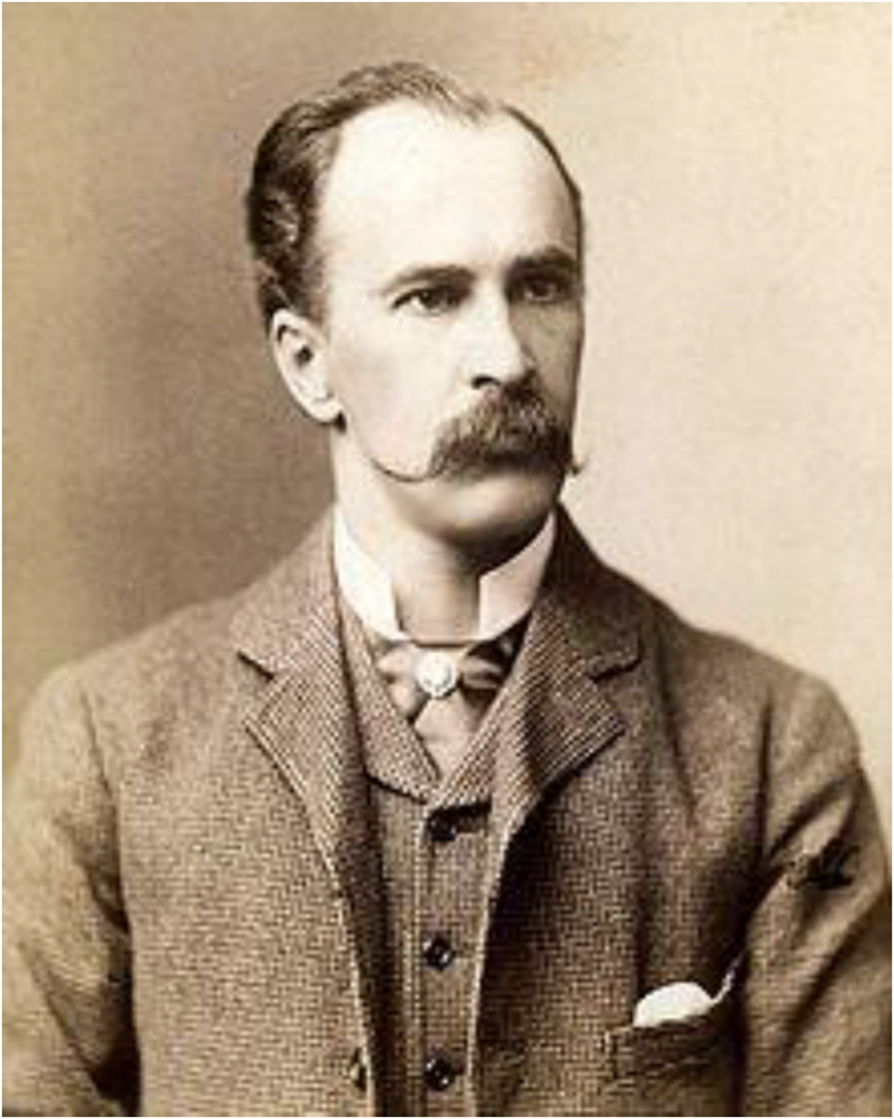
Sir William Osler

One can argue that it has never been easier to publish a case report, as the form has seen a resurgence in recent years. In the past, print journals were confined by limited space, but in the internet era of paperless journals, no such space restrictions exist. Additionally, the large volume of reports in digital format are easy to search, which is a key factor in their utility. Many case reports, by nature, have a narrow focus; indexing and online keyword searching allow simple retrieval of needles in a haystack [1].

Another reason for the upswing in case report publishing lies in the growing awareness that many aspects of clinical medicine cannot be addressed by large standardized trials. Large trials with validated data do much to advance medical science but are not suitable for all areas of medical research [3]. Neurology, for example, encompasses many conditions that have few patient representatives. Unlike specialties that routinely randomize thousands of patients in blinded, controlled studies, investigations of rare neurological diseases do well by obtaining cohorts of much fewer patients. Case reports in neurology prove even more valuable for this reason. The *Journal of Medical Case Reports* (JMCR) publishes reports by authors from many specialties, and at the time of this writing neurology publications were the fourth highest in number (*n* = 373) behind surgery (*n* = 875), cancer (*n* = 601), and medical imaging (*n* = 414). Neurology reports accounted for 5.4% of the total of 6872 case reports in all subject areas. *BMC Neurology* (the BioMed Central [BMC] flagship neurology journal) has published 23 case reports in 2015 as of this writing, around 19% of the total publications.

Publishing case reports makes good practical sense. It is comparatively easy for an individual physician or clinician to publish a case report rather than head a multi-institutional, transnational, multi-disciplinary trial that could take several years to decades to complete.

In approaching a neurology case report project, the most difficult decision is whether the case report is worth submitting for publication. Uniqueness goes a long way toward meeting the criteria for publication. Discussions with colleagues and research of the literature and case report databases help ascertain the extent to which a case is unique [4]. It may be helpful to keep in mind that uniqueness has its limits. Slight variations of a previously reported phenomenon, while technically unique, may not add much value. For example, drug reaction reports have value, but reporting an adverse reaction to a drug that is a member of a class of drugs known to produce such effects may not be informative or useful in general. A new or unusual location for a previously recognized disease may be useful to report only if accompanied by previously undocumented symptoms or if it required a particularly lengthy or costly diagnostic process [5].

The widespread notion that only very rare or novel cases meet the criteria for publication is mistaken. On the contrary, more frequently encountered scenarios prove useful: a common case that presents in an unusual way, or a common management strategy that faces new obstacles or unexpected outcomes, provides useful insights as much as do descriptions of exceedingly rare conditions [6].

Case reports that present valuable lessons are worth publishing. Those that increase awareness of lesser-known conditions, suggest an effective diagnostic strategy, or describe a more cost-effective approach to management are interesting. If a case sheds light on neuropathogenesis by illustrating a new theory or calling into question a current theory, the report may serve to spark further research [7]. In identifying emerging neurologic pathologies, case reports are the harbingers of the future.

Case reports provide an ideal venue for student and resident education, as the brief clinical caveats complement textbook reading. A good story stays with you for life. Many people can remember abstract medical concepts more easily when the information is linked to a patient. A case report can bring together different areas of medicine, such as neurology, neurophysiology, neuropathology, neuropharmacology, and neuroanatomy, illuminating a more holistic view of medicine.

Academic tertiary centers see patients referred for a second opinion who often present with challenging scenarios. Patients represent a variety of complex situations, including those who are difficult to diagnose, undiagnosed despite extensive work-up, in need of diagnosis confirmation (often in more serious diseases), or with requests for treatment and management recommendations. On many occasions, the diagnosis has previously been missed or misdiagnosed by physicians who have already seen the patient. Sometimes this is owing to the nature of the disease, as the pathology may not have declared itself fully. In these instances, the greatest teaching opportunities arise from mentors’ sharing their experience with trainees. Tips and clues to recognizing disease states and avoiding misdiagnosis, as well as instruction on how to institute therapy if the disorder is treatable, are important as lessons to be learned from challenging cases. A missed treatment, a treatment that is delayed for a critical period of time, or an inappropriate therapy can have significant deleterious effects on the course of disease and prognosis. The importance of taking a good history and performing a thorough neurological examination for accurate localization are key elements in arriving at a differential diagnosis before embarking on understanding pathogenesis or arriving at an etiology.

Many of these cases prove educational and are worth publishing. Writing a case or series reinforces many aspects of the training process, and house staff are encouraged to review cases, collect a series if there are similarities between cases (often discovered by retrospective record review), and try to get the report published. The process adds to training, as it entails thorough review of the cases, including the initial presentation that led to a delayed diagnosis or missed diagnosis; the data generated, such as electrophysiological testing in patients with neuromuscular disease and other diagnostic neuromuscular studies; a literature review; and treatment guideline recommendations. The writing process helps develop skills and knowledge in a neurologic subspecialty.

Several online journals are dedicated solely to publishing case reports. Table 1 lists some examples of online, open-access, peer-reviewed journals dedicated to case reports. *Case Reports in Neurology* is the only one we are aware of that is dedicated only to neurology case reports. BMC acknowledges the importance of case reporting and provides several platforms for publication. JMCR, a BMC publication, publishes high-quality case reports that contribute to the expansion of medical knowledge. Founded in 2007 by Michael Kidd and colleagues, the journal was believed to be the world’s first international medical journal devoted to publishing case reports in all clinical disciplines. *BMC Research Notes* (*BMCRN*), an online open-access journal with a slightly broader scope, invites submissions for case reports and series, short publications, and incremental updates to previous work. *BMCRN* hopes to add value to the research community by ensuring that such incremental research results do not remain unpublished in the absence of a sufficiently complete story required for a full article. *BMC Neurology* considers neurology case report submissions and articles on all aspects of the prevention, diagnosis, and management of neurological disorders, as well as related molecular genetics, pathophysiology, and epidemiology.

**Table 1:** Journals dedicated to case reports

Journal	Publisher	Year of inception	Website
*Journal of Medical Case Reports*	BioMed Central	2007	www.jmedicalcasereports.com
*BMJ Case Reports*	BMJ	2008	www.casereports.bmj.com
*Case Reports in Neurology*	Karger	2009	www.karger.com/journal/home/238704
*American Journal of Case Reports*	International Scientific Information	2008	www.amjcaserep.com
*Clinical Case Reports*	Wiley Online Library	2013	www.onlinelibrary.wiley.com/journal/10.1002/(ISSN)2050-0904
*International Medical Case Reports Journal*	Dove Press	2008	www.dovepress.com/international-medical-case-reports-journal-journal
*Oxford Medical Case Reports*	Oxford Journals	2014	www.omcr.oxfordjournals.org/
*Journal of Medical Cases*	Elmer Press	2010	www.journalmc.org
*International Journal of Case Reports and Images*	Edorium Journals	2011	www.ijcasereportsandimages.com

All of the listed journals are published online, open access, and peer-reviewed

Manuscripts submitted to *JMCR* and *BMC Neurology* must meet at least one of the following criteria [8, 9]:

- Unreported or unusual side effects or adverse interactions involving medications
- Unexpected or unusual presentations of a disease
- New associations or variations in disease processes
- Presentations, diagnoses, and/or management of new and emerging diseases
- An unexpected association between diseases or symptoms
- An unexpected event in the course of observing or treating a patient
- Findings that shed new light on the possible pathogenesis of a disease or an adverse effect

*BMC Neurology* states that case reports “should make a contribution to medical knowledge and must have educational value or highlight the need for a change in clinical practice or diagnostic/prognostic approaches” [9]. *BMCRN* states that it “considers medical case reports that describe any clinical case. Case reports submitted to *BMC Research Notes* do not need to be novel, but must be authentic cases and have some educational value” [10]. None of the three journals considers case reports describing preventive or therapeutic interventions, which generally require stronger evidence.

Common pitfalls of the case report process include proceeding with a case that is too similar to a previously reported case. Another is writing a manuscript that is too long. It is important to include enough information for the reader to identify similar patients and make an informed assessment, but only salient details. Extraneous information lengthens the report and confuses the message. Submitting a report geared to the wrong audience will prove unsuccessful. A thorough review of a journal’s author instructions as well as the sample articles provided can give a good indication of the publication’s readership. Care should be taken to avoid sweeping generalizations and statements of broad clinical implications based on one or two patients.

Some argue that case reports lack the scientific rigor of large, well-controlled studies, ranking at the bottom of the level of evidence hierarchy [11]. Given that case reports lack measures of statistical validity that come with large numbers of patients and controls, what is their role in this era of evidence-based medicine? Even for questions with relatively narrow focus, researchers in large studies struggle to account for multiple interacting components that contribute to experimental and control interventions. Every patient is unique, and the amount of flexibility in tailoring interventions, as well as the degree of variability in outcomes, defies simple descriptions put forth in experimental models [3].

Results of studies with large patient populations tend to average out individual variation, but it is in the variation that case reports illuminate concepts. The case report format allows investigation of the many factors that, in combination, form an outcome. Such multifactorial analysis is impossible in larger studies. Research areas that typically present such complexities, and in which case studies may help advance knowledge, include characterizing the interdependence of sequential events, understanding the impact of variability in treatment regimens, recognizing concomitant vs. causal comorbidities, and mapping contingency decision making [3].

The medical community benefits from descriptions of single cases, especially the unusual ones. Case reports may provide insight into particularly challenging conditions that physicians encounter and lead to better patient care, as well as further research into the discovery of new therapies.
